# Artificial intelligence‐assisted endoscopy changes the definition of mucosal healing in ulcerative colitis

**DOI:** 10.1111/den.13825

**Published:** 2020-10-08

**Authors:** Hiroshi Nakase, Takehiro Hirano, Kohei Wagatsuma, Tadashi Ichimiya, Tsukasa Yamakawa, Yoshihiro Yokoyama, Yuki Hayashi, Daisuke Hirayama, Tomoe Kazama, Shinji Yoshii, Hiro‐o Yamano

**Affiliations:** ^1^ Department of Gastroenterology and Hepatology Sapporo Medical University School of Medicine Hokkaido Japan

**Keywords:** artificial intelligence, histological healing, mucosal healing, red density system, ulcerative colitis

## Abstract

The relevance of endoscopic monitoring of ulcerative colitis (UC) has been translated into the new concept of “mucosal healing (MH)” as the therapeutic goal to achieve because a large amount of scientific data have revealed the favorable prognostic value of a healed mucosa in determining the clinical outcome of UC. Recent interest in MH has skewed toward not only endoscopic remission but also histological improvement (so called histological MH). However, we should recognize that there have been no prospectively validated endoscopic scoring systems of UC activity in previous clinical trials. Artificial intelligence (AI)‐assisted endoscopy has been developed for gastrointestinal cancer surveillance. Recently, several AI‐assisted endoscopic systems have been developed for assessment of MH in UC. In the future, the development of a new endoscopic scoring system based on AI might standardize the definition of MH. Therefore, “The road to an exact definition of MH in the treatment of UC has begun only now”.

## Introduction

Inflammatory bowel diseases (IBD) comprise the two major forms of chronic digestive inflammation: Crohn’s disease (CD) and ulcerative colitis (UC).^1^ Both are immunopathogenic complex diseases in which, on the basis of genetic susceptibility of the host, an excessive mucosal immune response against the complex enteric microbiota plays a role in the initiation and perpetuation of intestinal inflammation.^2^, ^3^, ^4^ Gastrointestinal (GI) endoscopy is clinically important for the management of IBD patients because of its involvement in the diagnosis of UC and CD, in monitoring the response to anti‐inflammatory therapy, and dysplasia surveillance. Many physicians have understood that mucosal healing (MH) is a key clinical treatment goal in IBD patients.^5^ Since it has been shown that complete MH is one of the most crucial aspects that predicts sustained clinical remission, the need for hospitalization and the probability of surgery‐free survival among patients, a precise and detailed real‐time assessment of the mucosal surface has become more important than ever for the medical management of IBD patients.^6^, ^7^, ^8^ In this context, advanced endoscopic imaging techniques including endocytoscopy (EC; Olympus, Tokyo, Japan) and confocal laser endomicroscopy (CLE) have been applied to allow precise, ultrastructural and even microscopic characterization of the inflammation of UC.^9^ However, even if we used these modalities to assess MH in UC, the interpretation of the gained findings was operator‐dependent. Artificial intelligence (AI)‐assisted endoscopy has been developed for evaluation of endoscopic activity of UC. In this article, we review the current literature on AI‐associated endoscopy regarding the assessment of MH with histological remission in UC patients.

## What is machine learning, deep learning, and artificial intelligence?

### Machine learning

**Figure 1:**
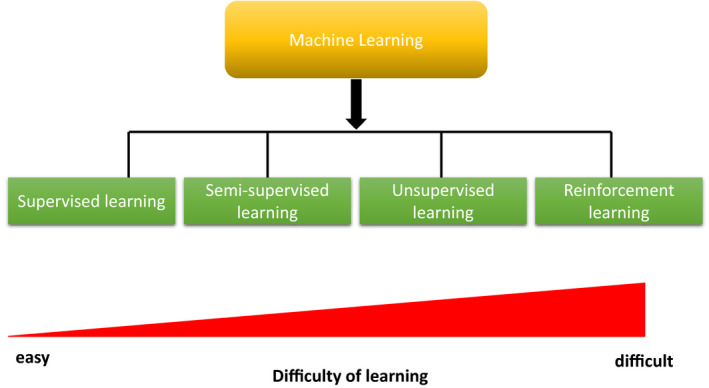
Four kinds of algorithm regarding machine learning with the difficulty of learning.

In 1959, Samuel defined machine learning as the “field of study that gives computers the ability to learn without being explicitly programmed”.^10^ There are four types of algorithms involved in machine learning (Fig. 1). (i) Supervised learning: This is a method of training the computer by labeling the training data tagged with the correct answer. (ii) Unsupervised learning: This is a method of training without marking the training data. (iii) Semisupervised learning: This method utilizes a large amount of unlabeled data based on a small amount of labeled data, making it easier to train the model (computer). (iv) Reinforcement learning: In this method, the network learns behaviors to maximize the “rewards” (scores) set as objectives in a given “environment”.^11^, ^12^, ^13^

### Neural network system

A neural network is a system that imitates the mechanism of human nerve cells (neurons). By using neural networks in multiple layers, it becomes possible to learn deeper features contained in the data in a stepwise fashion. By inputting a large amount of image, text, voice and other kinds of data into a multilayered neural network (convolutional neural network, CNN), the computer model automatically learns the features contained in the data in each layer. Deep learning (DL) is based on neural network systems. These structures are unique to DL, making DL models accurate and sometimes more accurate than humans.

### Deep learning

**Figure 2:**
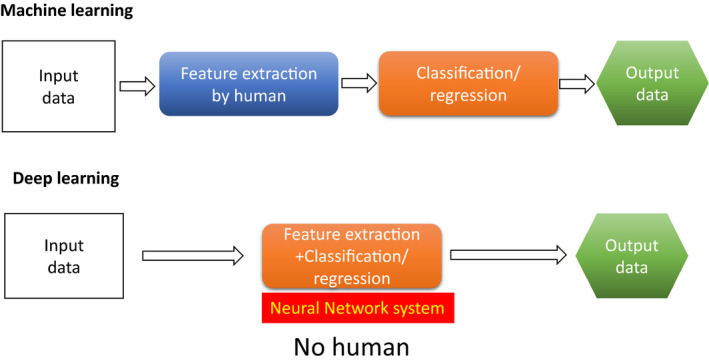
The difference between traditional machine learning methods and deep learning.

Deep learning is a machine learning method that allows a computer to learn the tasks that humans naturally perform.^14^ It is a technology that supports the rapid development of the application of artificial intelligence (AI) to practical tasks in many fields. For example, DL is the key to self‐driving cars and voice recognition. DL has been gaining attention recently because DL is becoming capable of achieving high levels of performance. The difference between traditional machine learning methods and DL is whether or not a human feature extraction is required (Fig. 2).^15^, ^16^

### Artificial intelligence

**Figure 3:**
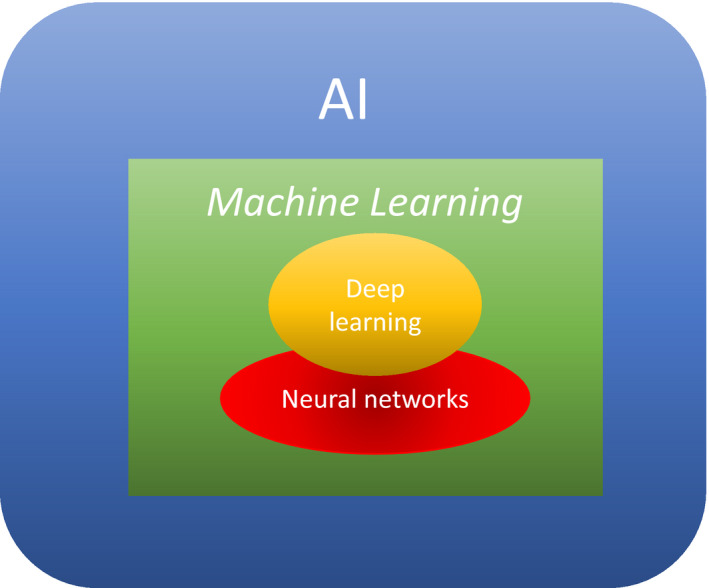
The relationship between deep learning and machine learning/artificial intelligence.

Artificial intelligence involves a computer performing intelligent tasks that are normally performed by humans, such as learning and problem‐solving.^16^, ^17^ There are two types of AI; general‐purpose AI and specialized AI. Briefly, AI that solves various problems in different domains is called general‐purpose AI. AI specializing in individual domains to demonstrate its capabilities is called specialized AI. For example, there is AI that specializes in voice recognition, and AI specialized in Go or Shogi. Figure 3 shows the relationship between DL and AI/machine learning.

## Mucosal healing in UC – the subjective endoscopic score as the main stay of MH assessment

The concept of MH was introduced approximately 50 years ago.^18^ In a clinical practice, physicians have accepted the definition of MH as “the complete resolution of the visible alterations or lesions, regardless of their severity and/or type at baseline colonoscopy”. Although there have been 50 years of clinical trials and endoscopic scoring systems with different designs have been developed (Baron score, Mayo score, Sutherland, Powell‐Tuck and Rachmilewitz indices, among others),^19^, ^20^, ^21^, ^22^, ^23^, ^24^, ^25^, ^26^ the definitions and scoring methods of these instruments have never been prospectively validated. Two new scoring systems were developed and prospectively validated: the Ulcerative Colitis Endoscopic Index of Severity (UCEIS) and the Ulcerative Colitis Colonoscopic Index of Severity.^27^, ^28^ Nevertheless, no validated endoscopic score for the evaluation of UC activity evaluation reflects the complexity.

## Histological MH – is this an ideal therapeutic goal?

The necessity of the histological evaluation of the colonic mucosa of UC patients has been emphasized. Bessissow *et al*.^29^ reported that the presence of basal plasmacytosis predicts UC clinical relapse in patients with complete MH. Peyrin‐Biroulet *et al*.^30^ indicated that data indicating a prognostically relevant role for histologic activity in the mucosa of UC patients, and that finding, in addition to the macroscopic activity, has opened the door to the concept of “histological MH”, with the complete absence of clinical, laboratory, endoscopic and histological features of active inflammation. However, it remains unclear whether the histological results of rectal biopsy reflect inflammatory conditions in the entire colonic mucosa of patients with extensive UC who have achieved endoscopic remission.

## Emergence of confocal laser endomicroscopy and endocytoscopy for the assessment of mucosal inflammation in UC

New endoscopic techniques such as in vivo confocal endomicroscopy (CLE), focus on histologic assessment. CLE allows the in vivo microscopic imaging of cellular and subcellular structures at approximately 1000×‐fold magnification. This intriguing technique has the capability to obtain “real‐time” histology‐like images of the GI mucosa. Several reports have demonstrated that CLE enables us to identify the different features of colonic crypts in the inflamed and noninflamed rectal mucosa in UC patients and to visualize bacterial translocation and abnormal epithelial barrier function.^31^, ^32^, ^33^ Thus, CLE can contribute to the understanding of the pathophysiology of UC. Endocytoscopy (EC) is based on the principle of contact light microscopy. EC systems are either integrated into the distal tip of a standard endoscope (iEC) or probe‐based (pEC).^34^, ^35^ For example, pEC visualizes architectural details, cellular features, and vascular pattern morphology at a magnification of up to 1390×. Bessho *et al*.^36^ reported the correlation of assessment of microscopic features in UC patients between EC and conventional histopathology. Interestingly, a scoring system based on EC findings was strongly correlated with Matts’ histological grades. Taken together, these findings show that both CLE and EC have strong potential for identifying subtle mucosal inflammation in real time by identifying single mucosal inflammatory cells in conjunction with architectural changes. On the other hand, we must acknowledge that the mucosal inflammation detected by CLE and EC only reflects a part of the histological condition in the colonic mucosa of UC alone because the observational range of intestinal mucosa for these modalities is very limited.

## Importance of AI‐assisted endoscopy for evaluating mucosal inflammation

Image recognition using AI techniques can be used to grade endoscopic images and morphology. AI systems allow us to make assessments with lower bias and more objective interpretation methods not only for GI cancer but also for IBD. As mentioned earlier, objective treatment targets are required in a treat to target (T2T) approach for UC. Many physicians hope for the development of an automated endoscopic scoring system that correlates with histology because the AI‐assisted evaluation of mucosal inflammation can serve as an objective predictor of sustained remission in UC. Currently, a computer‐aided diagnosis (CAD) system, has been applied to the objective assessment of mucosal inflammation in UC. The characteristics of AI‐assisted endoscopy are shown in Table 1.

**Table 1:** The characteristics of artificial intelligence‐assisted endoscopy

	Red density^44^	MAGIC score^43^	CAD system for Mayo score^37^	Endo brain^39^	Fujifilm research^40^ (estimation mucosal capillary vessel)	Sony research^41^	Michigan research^42^
Range of assessment of mucosal inflammation^†^	Large	Large	Large	Small	Small	Large	Large
Scoring system	Yes	Yes	No	Yes	No	Yes	Yes
Correlation with histology	Yes	Yes	No	Yes	Yes	Yes	No

^†^ Large: whole assessment of inflammation in colonic mucosa on image. Small: partial assessment of inflammation in colonic mucosa on magnified image.

### Computer‐aided diagnosis of endoscopic activity in UC

Even if endoscopists experienced the evaluation of the inflammatory activity in UC, interobserver variability still exists. Ozawa *et al*.^37^ reported the construction of a CAD system using a CNN, and the performance of the system was evaluated by tagging a large dataset of endoscopic images from patients with UC. They constructed this CNN‐based CAD system based on GoogLeNet architecture. The data used to train the CNN were 26,304 colonoscopic images from a cumulative total of 841 patients with UC. These images were tagged with Mayo endoscopic scores (MESs). The performance of the trained CNN with regard to identifying normal mucosa (MES 0) and mucosal healing mucosa (MES 0‐1) was evaluated in an independent test set of 3981 images from 114 patients with UC by calculating the areas under the receiver operating characteristic curves (AUROCs). As expected, this CNN‐based CAD system had excellent performance, with AUROCs of 0.86 and 0.98 for the identification of MES 0 and 0‐1, respectively. The authors stated that the CNN‐based CAD system would support less‐experienced endoscopists and reduce interobserver variability with regard to the MES. Unfortunately, this CAD system was not tagged with the histological findings.

### EndoBRAIN system (EndoBRAIN; Cybernet Systems, Tokyo, Japan)

Mori *et al*. reported the usefulness of a CAD system for EC imaging (EC‐CAD) to allow untrained, nonexpert endoscopists to derive benefits from the high diagnostic performance of EC. This system provides fully automated instant classification of colorectal polyps during routine colonoscopy.^38^ Maeda *et al*.^39^ applied this CAD system to develop and evaluate the prediction of histologic inflammation in UC patients. They retrospectively reviewed the data of 187 patients with UC from whom biopsy samples were obtained after EC. All EC images were tagged with reference to the histologic activity observed in the biopsy samples. They validated 525 sets of 525 independent segments, which were collected from 100 patients. Machine learning was applied to 12,900 EC images from the remaining 87 patients and were used to construct the CAD system. This CAD system was shown to have a diagnostic sensitivity, specificity, and accuracy of 74% (95% confidence interval (CI), 65–81%), 97% (95% CI, 95–99%), and 91% (95% CI, 83–95%), respectively. The authors concluded that the EC‐CAD system could contribute to the fully automated identification of the persistent histologic inflammation associated with UC. However, we should keep in mind that the inflammation of the colonic mucosa in UC is not uniform, but heterogeneous. The images captured by the EC‐CAD system does not necessarily reveal the complete circumferential colonic inflammatory status.

### Histological evaluation based on mucosal capillary pattern in UC by the CAD system

It is recognized that the infiltration of neutrophils is associated with irregularities in the pericryptal capillaries in the inflamed mucosa. Bossuyt *et al*.^40^ developed an objective automated endoscopic tool to assess histological remission by focusing on the evaluation of the morphology of the pericryptal capillaries. The algorithm included two steps. First, bleeding (mucosal/luminal) was assessed by pattern recognition (number of pixels: bleeding (NPBL)). Samples with bleeding (high NPBL) were automatically classified as nonremission. Second, in the case of nonbleeding samples (low NPBL), the degree of congestion of the capillaries (number of pixels: high density (NPHD)) was measured to assess an ideal cut‐off value to identify histological remission (Geboes score (GBS) <2B.1; no neutrophils in the lamina propria). This algorithm for automated image analysis detected histological remission with a higher performance (sensitivity 0.79, specificity 0.90) than the UCEIS (sensitivity 0.95, specificity 0.69) and MES (sensitivity 0.98, specificity 0.61), resulting in positive predictive values of 0.83, 0.65 and 0.59 for the algorithm of automated image analysis algorithm, UCEIS and MES, respectively. Mucosal capillary pattern recognition based on an automated image analysis with short‐wavelength monochromatic light detects histological remission with high accuracy in UC patients. The authors concluded that this technique provides an objective and quantitative tool for the assessment of histological remission in UC. However, the NPHD value only reflects the grade of mucosal inflammation in a limited part of the colonic mucosa, as in the EndoBRAIN system.

### Deep neural network for grading the endoscopic disease severity of UC

Takenaka *et al*.^41^ reported the development of a deep neural network system for the consistent, objective, and real‐time analysis of endoscopic images from patients with UC. They constructed a deep neural network for the evaluation of UC (DNUC) algorithm with reference to 40,758 images of colonoscopies tagged with 6885 biopsy results. The accuracy of the DNUC algorithm was validated in a prospective study of 875 patients with UC who underwent colonoscopy from April 2018 to April 2019, with 4187 endoscopic images and 4104 biopsy specimens. They defined endoscopic remission (ER) as a UCEIS of 0; and histologic remission as a Geboes score of three points or less. The results of this study demonstrated that the accuracy ratio of identifying UC patients with ER by the DNUC algorithm was 90.1% accuracy (95% CI 89.2–90.9%) and the accuracy of identifying UC patients with histologic remission by the DNUC algorithm was 92.9% (95% CI 92.1–93.7%). Takenaka *et al*. concluded that the DNUC algorithm could provide objective, consistent and real‐time endoscopic evaluation. Additionally, they emphasized that the DNUC algorithm could be not only an alternative to a central reader in clinical trials but also a useful tool in the training of gastroenterologists for the assessment of endoscopic activity of UC. This is a novel system of evaluation of circumferential mucosal inflammation in UC, therefore, we strongly hope that this system will become part of routine clinical practice. On the other hand, a multicenter prospective study is needed to confirm the accuracy of this system because the DNUC algorithm was constructed with data from a single center.

### Application of CNN to generate DL models for grading the endoscopic disease severity of UC

Stidham *et al*.^42^ investigated the feasibility of generating a DL algorithm to grade the endoscopic severity of UC and applied it to full‐motion video recordings of colonoscopies. Based on data from Michigan’s endoscopic imaging database (16,514 colonoscopic images in 3082 UC patients) and the university’s electronic health records, they constructed a 159‐layer CNN as a DL model to train and categorize images into two groups: ER (defined by MES 0 or 1) and active disease (MES 2 or 3). They showed that this CNN could distinguish ER from active disease with an AUROC of 0.966 (95% CI, 0.967–0.972); a positive predictive value (PPV) of 0.87 (95% CI, 0.85–0.88) with a sensitivity of 83.0% (95% CI, 80.8–85.4%), and a specificity of 96.0% (95% CI, 95.1–97.1%); and a negative predictive value (NPV) of 0.94 (95% CI, 0.93–0.95). Applying the CNN to entire colonoscopy videos had similar accuracy for identifying moderate‐to‐severe disease (AUROC, 0.97; 95% CI, 0.963–0.969). Thus, the performance of DL models to approximate the disease assessment made by experts has excellent reproducibility, objectivity, and speed. However, a limitation of this study was that they did not construct the system based on histological data. In addition, they did not differentiate between MES 0 and 1. Therefore, we hope that a more precise DL model will be developed that can differentiate MES 0 and 1 based on tagged histologic results because several studies have highlighted the distinct difference in the relapse rate between clinically quiescent UC patients with MES 0 and those with MES 1.

### Development of the MAGIC score for the assessment of mucosal inflammation

**Figure 4:**
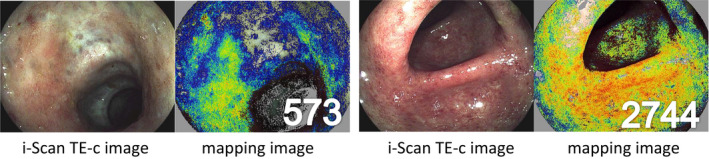
The representative images of MAGIC scores. i‐Scan TE‐c images (left) and mapping images (right). As the degree of inflammation increased, the calculated score of the mapping image increased.

The assessment of a MES of 0 or 1 varies widely between endoscopists. Additionally, several studies have shown that endoscopic MH does not necessarily reflect quiescent microscopic UC disease activity. It should be emphasized that no scoring system based on the combination of endoscopic images and histology has been developed for clinical use. If we can develop a scoring system reflecting mucosal inflammation or specifically reflecting MH in UC, it will be ideal from the perspective of the current T2T concept. We retrospectively reviewed the data from 52 UC patients with clinical remission who had undergone routine colonoscopy with the i‐scan TE‐c system, which can enhance images of normal or inflamed colonic mucosa with improved color contrast.^43^ With reference to images captured by the i‐scan TE‐c system, the degree of inflammation was quantified for the entire screen by correlating the value with the reference value for each pixel in the HSV color space. Based on the data, we developed the Mucosal Analysis of Inflammatory Gravity by i‐scan TE‐c Image (MAGIC) score by supervised machine learning, which was defined as the mean value of the quantified values for each pixel. Representative images of MAGIC score are shown in Figure 4. The associations among the MAGIC score, MES, and histologic activity (Geboes score) were investigated. The MAGIC score was significantly higher in the MES 1 group than in the MES 0 group (779.8 ± 488.4 vs. 487.2 ± 378.2, *P* = 0.0034) and was significantly correlated with the Geboes score (*P* = 0.015). Interestingly, even UC patients with MES 0 have widely ranging MAGIC scores, as do those with endoscopic MES 1. These data suggested that the stratification of mucosal inflammation in UC by the MAGIC scoring could be possible among UC patients with MESs of 0 or 1.

### Evolution of the MAGIC score: construction of the red density system

Using the MAGIC score as a foundation, we developed an operator‐independent computer‐based tool to determine UC disease activity based on endoscopic images, which we named the “Red density (RD) system”.^44^ First, a computer algorithm using data from 29 consecutive patients with UC and six healthy controls was constructed by supervised machine learning.

**Figure 5:**
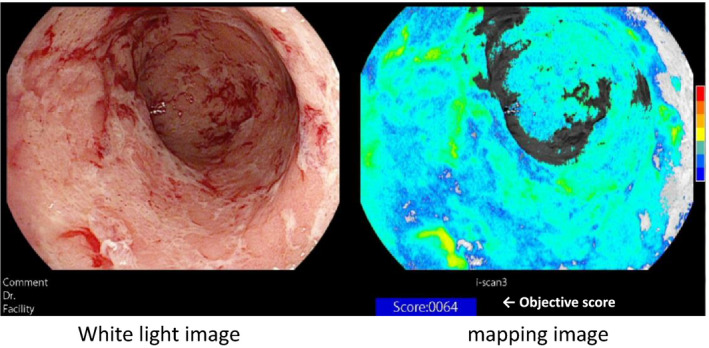
Red density (RD) image and score displayed on the monitor.

Red density was determined using normal images that were taken with a white light source and without magnification. The RD system was based on the red channel of the red‐green‐blue pixel values and pattern recognition from endoscopic images. The algorithm was refined in sequential steps to optimize the correlation with endoscopic and histological disease activity. Second, the operating properties were tested in patients with UC flares necessitating treatment escalation. To validate the algorithm, we tested the correlations between the RD system score and clinical, endoscopic and histological features in a validation cohort. Multiple regression analysis was used to assess the correlation of the data with Robarts histological index (RHI). The display of the RD system during endoscopy can be seen in the supplementary video (Fig 5, Video S1). We found that the RD score correlated with RHI (*r* = 0.74, *P* < 0.0001), the MES subscores (*r* = 0.76, *P* < 0.0001) and the UCEIS (*r* = 0.74, *P* < 0.0001). Thus, the RD system is a novel modality that provides an objective computer‐based score that accurately assesses disease activity in UC. Currently, we are starting a larger, prospective study to confirm the predictive value and accuracy of the RD system score for MH. Currently, Pentax is seeking regulatory approval for the RD system and plan to hold meetings with the regulatory authorities before submission or registration.

## Role of AI‐assisted endoscopy for the surveillance of colitis‐associated cancer

In addition to the assessment of MH in UC, the primary clinical issue is the surveillance of colitis‐associated cancer (CAC). Chronically sustained inflammation triggers colitis‐associated lesions, which tend to present in a flat or multifocal pattern.^45^ Colitis‐associated dysplasia and cancer have pathologically distinct patterns from sporadic adenoma/carcinoma. It is challenging to distinguish the morphological features of these lesions from the surrounding inflamed mucosa with standard white light endoscopy.^46^ There are multiple new endoscopic imaging technologies, including standard chromoendoscopy with indigo carmine or methylene blue, virtual chromoendoscopy narrow‐band imaging, Fujinon intelligent color enhancement, and i‐Scan, autofluorescence imaging, CLE and EC, that have been applied to identify CAC.^47^ However, there are few reports on the application of AI‐assisted colonoscopy techniques for the diagnosis of CAC. Most recently, Fukunaga *et al*.^48^ reported the usefulness of the EndoBRAIN system for the diagnosis of high‐grade dysplasia in a patient with a long‐term history of UC. They concluded that the EndoBRAIN system could help nonexpert endoscopists identify CAC, thereby avoiding unnecessary biopsies. This case report strongly indicates the significance of EC for the diagnosis of neoplastic lesions associated with UC and sporadic neoplasia. However, there are still a small number of images of colitis‐associated dysplasia and cancer in UC that have been histologically tagged. A large amount of imaging data on dysplasia and CAC in UC patients and validation is needed to improve the diagnostic performance of the EndoBRAIN system.

## Limitation of AI for the assessment of mucosal inflammation in UC

We recognize that a deeper model with a large number of parameters tends to be more accurate, and accuracy is also improved by combining the predictions of multiple models (ensemble) rather than using the predictions made by a single model. Based on these principles, a large number of images with an accurate diagnosis regarding MH is required. As mentioned earlier, many researchers have utilized a large number of endoscopic pictures evaluated by the MES, and UCEIS and tagged with the histologic results to develop an AI model for the assessment of intestinal inflammation in UC. However, there is still a problem because MH in UC lacks an exact, correct definition. Since DL is automatic, the content and direction of the learning change considerably depending on the data provided. Therefore, it is essential to select the data to be read carefully. In this regard, it remains unclear whether the present endoscopic images, endoscopic index and histologic score are suitable parameters for DL. At present, if we establish a more accurate AI model for the assessment of MH, endoscopic images combine with biological parameters such as the levels of fecal calprotectin and leucine‐rich α2 glycoprotein should be used. The development of a graphic processing unit can contribute to the improvement of the recognition of details of endoscopic images; however, this may not be enough. The addition of molecular analysis data, such as cytokine and epithelial regenerative molecule expression in the intestinal mucosa of patients with long‐term quiescent UC, as parameters for DL in the future may enable the precise identification of MH.

## In Summary

Since it is suggested that a T2T algorithm could improve outcomes in the long term in UC, the intra‐ and interobserver variations in endoscopic assessment of UC are a clinical issue. However, the development of AI‐assisted endoscopy has led to growing interest in the use of new imaging technologies, and opened a new path toward not only the diagnosis of GI cancer but also the IBD field. Now, we are seeing AI being used in medical image interpretation to improve objectivity and reproducibility and there is a possibility that AI‐assisted medical imaging can broaden access to expert‐level of assessments or exceed it. There may even be a time when we do not grade or diagnose endoscopic images may come in near future. As AI systems contribute to the exact diagnosis and treatment of human disease, we should continue to learn best practices in health care.

## Conflict of Interest

Professor. Nakase was supported by grants or donations from Abbvie Inc., Kissei Pharmaceutical Co., Ltd., Kyorin Pharmaceutical Co.,Ltd., Mitsubishi Tanabe Pharma Corporation, Janssen Pharmaceutical K.K, Takeda Pharmaceutical Co.,Ltd., Pfizer Lnc., Cell gene Corporation., EA Pharma Co.,Ltd., Zeria Pharmaceutical CO.,Ltd., Mochida Pharmaceutical Co.,Ltd., Nippon Kayaku Co.,Ltd., Daiichi Sankyo Company, Limited., JIMRO Co.,Ltd., Hoya Group Pentax Medical, Boehringer Ingelheim GmbH, and Bristol‐Myers Squibb Company. Other authors have no COI to disclose.

## Funding Information

This work was supported by Health and Labour Sciences Research Grants for research on intractable diseases from the Ministry of Health, Labour and Welfare of Japan (Investigation and Research for intractable Inflammatory Bowel Disease to HN) and Japan Society for the Promotion of Science (JSPS) Grants‐in‐Aid for Scientific Research (KAKENHI) Grant Number JP18H02799 (to HN).

## Supporting information


**Video S1** The RD image and score was seen in real time, along with the white light image.
